# Abnormal ankle-brachial index, cardiovascular risk factors and healthy lifestyle factors in hypertensive patients: prospective cohort study from a primary care urban population

**DOI:** 10.1186/s12875-022-01837-1

**Published:** 2022-09-09

**Authors:** Ana María Armas-Padrón, Miriam Sicilia-Sosvilla, Sergio Rodríguez-Bello, María Dolores López-Carmona, Pedro Ruiz-Esteban, Domingo Hernández

**Affiliations:** 1 La Cuesta Primary Healthcare Centre, La Laguna, E-38320 Tenerife, Spain; 2 Internal Medicine Department, Hospital Regional Universitario de Málaga, E-29010 Málaga, Spain; 3 Nephrology Department, Hospital Regional Universitario de Málaga, University of Málaga, Instituto de Investigación Biomédica de Málaga (IBIMA), REDinREN (RD16/0009/0006 and RICORS RD21/0005/0012), E-29010 Málaga, Spain; 4 Nephrology Department, Carlos Haya Regional University Hospital, Avda. Carlos Haya s/n., E-29010 Malaga, Spain

**Keywords:** Hypertension, Ankle-brachial index, Peripheral artery disease, Arterial stiffness, Cardiovascular health metrics, Cardiovascular risk factors

## Abstract

**Background:**

Peripheral arterial disease (PAD) and arterial stiffness (AS) may be hypertension-mediated vascular lesions. Both are determined by an abnormal ankle-brachial index (ABI) and are predictors of cardiovascular disease (CVD) and mortality. We assessed the relationship in urban hypertensive patients between an abnormal ABI and an ideal cardiovascular health (CVH) score, plus other healthy factors, with unfavourable outcomes.

**Methods:**

We studied 243 hypertensive patients from a primary care urban population, followed for two years. Clinical data, comorbid conditions, including hypertension-mediated organ damage (HMOD) and hypertension-related comorbidities (HRC), hospitalizations and mortality were also recorded.

**Results:**

A low prevalence of ideal CVH was observed in urban hypertensive patients. The ABI ≤ 0.9 group (*n* = 16) showed a higher proportion of prior CVD other than PAD, mortality and hospitalizations than the ABI > 1.4 group (*n* = 41), and a poorer lipid, metabolic and renal profile. An inverse relationship between CVH score and ABI ≤ 0.9 and unfavourable outcomes (HMOD, HRC, death or hospitalization) was observed. Chronic kidney disease (CKD) and diabetes were independently associated with an ABI ≤ 0.9. Age, sex, diabetes, CKD, ABI ≤ 0.9 and ideal cholesterol were also associated with outcomes, but not other CVH metrics.

**Conclusions:**

Besides a low prevalence of ideal CVH, an inverse relationship between CVH score and ABI ≤ 0.9 and unfavourable outcomes was observed in hypertensive patients from an urban population. Stronger efforts to promote ideal CVH may improve outcomes in this particular population.

**Supplementary Information:**

The online version contains supplementary material available at 10.1186/s12875-022-01837-1.

## Background

Hypertension is a well-known risk factor for cardiovascular disease (CVD) and both peripheral artery occlusive disease (PAD) and arterial stiffness (AS) are considered common targets of hypertension-mediated organ damage (HMOD), as well as important manifestations of systemic atherosclerosis leading to a poor outcome [1, 2].

The incidence of PAD and AS in the lower extremities is increasing with age worldwide, especially in people aged older than 65 years (20%), with many of these being asymptomatic and thus untreated [3, 4]. Additionally, both PAD and AS have been identified as robust predictors of future CVD events [5]. Approximately 40% of individuals with PAD have no concomitant other CVD (e.g. coronary artery disease or cerebrovascular disease), suggesting the need to study PAD in this setting particularly [6, 7].

The ankle-brachial index (ABI) is a simple, reliable, non-invasive way to diagnose PAD or AS, and predict CVD in the general population, as recommended in guidelines of the American College of Cardiology/American Heart Association Task Force [8]. In fact, the ABI measurement is a marker of systemic atherosclerosis and thus is associated with both atherosclerotic risk factors and prevalent CVD in other vascular beds in diverse populations, including hypertensive patients. Guidelines for the management of hypertensive patients categorize the cardiovascular risk of this particular population according to several factors, including blood pressure, asymptomatic organ damage, as well as the presence of diabetes, symptomatic CVD, or renal failure [9]**.** In this categorization, asymptomatic organ damage is assessed by the presence of asymptomatic PAD, defined as ABI values less than 0.9. This cut-off value has sensitivities ranging from 68–84% and specificities from 84–99% to diagnose PAD, according to the clinical guideline on the management of these patients [10]. Indeed, ABI ≤ 0.9, indicating PAD, is associated with many cardiovascular risk factors, including hypertension, diabetes, dyslipidaemia, smoking history, and other non-traditional cardiovascular risk factors, and implies a 3–4 times higher risk of cardiovascular mortality [11–14]. This index has also been used in combination with the Framingham risk function to predict stroke [15], and in a meta-analysis to improve cardiovascular and mortality risk prediction [5]. Additionally, an ABI > 1.4, indicating that the arteries are not able to be compressed, suggests AS, which is associated with a poor prognosis and a higher all-cause and cardiovascular mortality in both the general and the hypertensive population, as well as in renal patients [16–18]. The increased risk associated with a high ABI appears to be lower than in patients with a low ABI. In any case, the impact of a high ABI on risk stratification in hypertensive patients has not been sufficiently assessed.

In 2010 the American Heart Association developed a metric, termed Life’s Simple 7 (LS7), to define and promote ideal cardiovascular health (CVH) metrics, and which includes 4 ideal health behaviours and 3 ideal health factors [19]. Accordingly, several studies have indicated that a higher number of metrics fulfilling the ideal CVH definition was associated with a lower incidence of CVD, including subclinical PAD and AS, renal disease and all-cause mortality [4, 20–29]. Indeed, several studies have demonstrated the relationship and its predictive value between CVH metrics and PAD and AS, in different race/ethnic groups [26, 30]. While PAD shares multiple risk factors included in the LS7 with other CVDs, the specific association between these LS7 metrics and PAD and AS in hypertensive patients has not been fully assessed. In addition, hypertension-related chronic kidney disease (CKD) is a common and well-known risk factor for CVD, but this crucial risk factor is not included in the CVH metrics even though many of the individual components of the LS7 metrics are associated with CKD.

We hypothesized that an ideal cardiovascular health status is related to a lower risk of abnormal ABI (ABI ≤ 0.9 or > 1.4). Thus, we aimed to assess in a population-based hypertensive patient cohort from an urban primary care centre the association between the individual and combined factors that comprise the LS7, determined at baseline, and the presence of pre-existing abnormal ABI. Additionally, we also analysed the relationship between LS7 metrics and other HMOD and hypertension-related comorbidities (HRC), including CKD, recorded at baseline and during the two years of follow-up. Finally, we also studied the association between ideal CVH and several cardiovascular risk factors and unfavourable outcomes in this particular population.

## Methods

### Subjects

**Fig. 1:**
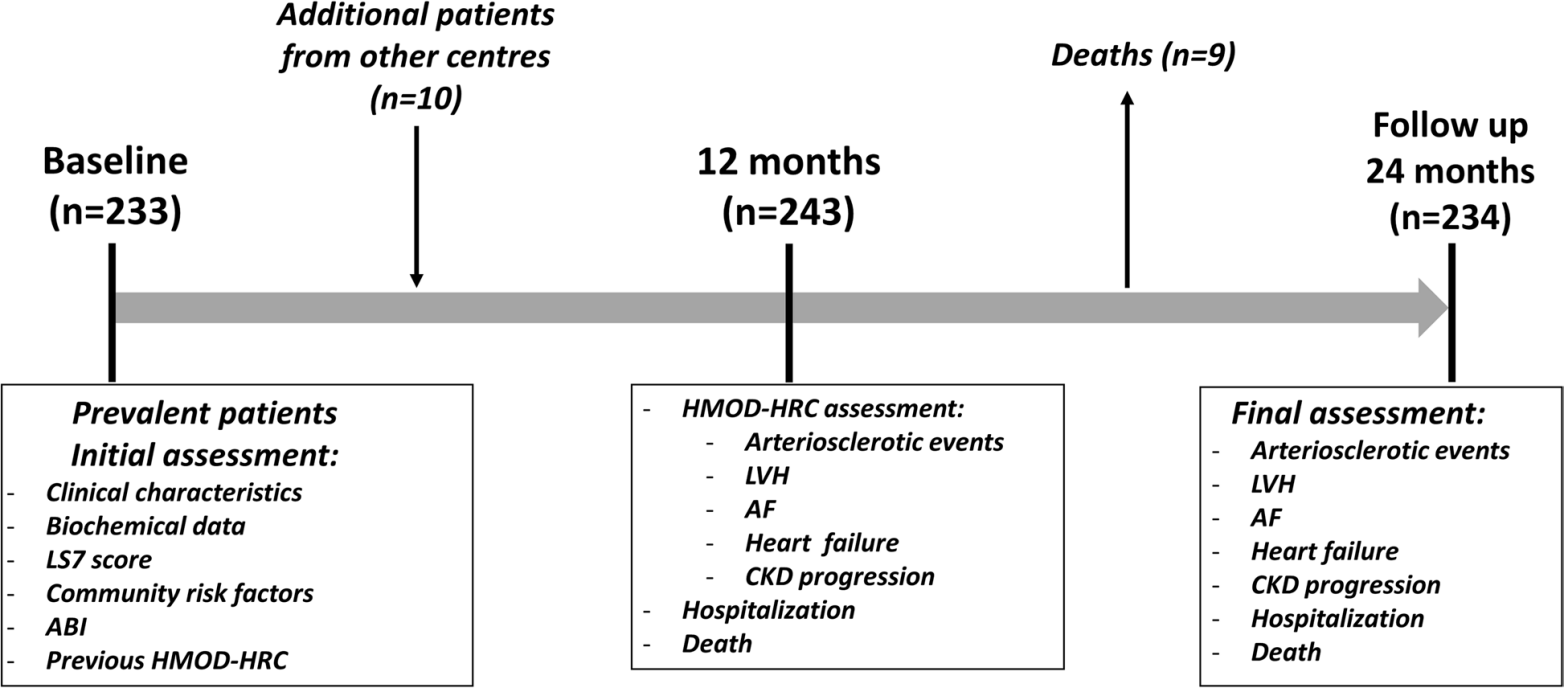
Study flowchart. Abbreviations: ABI, ankle-brachial index; AF, atrial fibrillation; CKD, chronic kidney disease; LVH, left ventricular hypertrophy; LS7, life’s simple 7; HMOD-HRC, hypertension-mediated organ damage and hypertension-related comorbidities

A total of 243 consecutive adult Caucasian hypertensive patients who were examined between September 2018 and September 2019 in an urban health care centre (La Cuesta, La Laguna, Tenerife, Spain) were enrolled in this study. During the recruitment period we included both prevalent hypertensive patients from our primary care centre and additional prevalent hypertensive patients referred from other care centres. All hypertensive individuals were previously diagnosed with primary hypertension and the median follow-up period was 156.5 months (interquartile range 84–204). Exclusion criteria were patients under 18 years of age, the presence of secondary hypertension and refusing informed consent. Follow-up was censored up to the end of the observation period (2 years after baseline assessment), or at the time of death. Thus, all surviving patients were followed for at least 24 months from their study inclusion. All patients gave their informed consent. The study protocol was approved by the institutional review board of a tertiary hospital in Spain (University Regional Hospital of Malaga) and is in accordance with the Declaration of Helsinki. Figure 1 shows the flowchart of the study.

### Data collection

Information collected included demographic and socioeconomic data, education level, the average family income, nocturnal work, marital status, smoking status, physical activity, alcohol consumption, quality of diet, salt intake, past medical history (myocardial infarction, stroke, atrial fibrillation, and PAD) and active treatment such as anti-hypertensive agents, hypoglycaemic agents and lipid-lowering agents, all collected using a standardized questionnaire. Smoking status was classified as ‘‘never’’, ‘‘former’’ or ‘‘current’’ and based on self-report. Alcohol intake status was as ‘‘never’’, ‘‘former’’ or ‘‘current’’. Drinking was defined as an average daily strong spirit (alcohol content > 50%) consumption of 100 ml or more for at least the previous year. Diet was defined according to American Heart Association criteria using 5 components of a healthy diet (high intake of fruits and vegetables, fish, whole grains; low intake of sodium and sugar-sweetened beverages). Diet was identified by self-reported questionnaire. An ideal diet was defined as meeting four or five of the following criteria: fish consumption ≥ 2 servings/wk, fruit/vegetables ≥ 4.5 cups/d, sodium intake < 1500 mg/d, sugar < 450 kcal/wk, and fibre/carbohydrate ratio > 0.1.

Physical activity level was categorized as: none, 1–3 times per week, and ≥ 4 times per week, obtained from questionnaires. Body mass index was calculated from height and weight measurements. Fasting blood glucose, resting blood pressure, serum cholesterol, renal function evaluated by MDRD equations and self-reported smoking status were obtained at the moment of inclusion in the study. Resting blood pressure measurement was obtained while sitting and relaxing for 3–5 min with the arm resting on the table with the mid-arm at heart level, the back supported on a chair, the legs uncrossed and the feet flat on the floor, in accordance with hypertension clinical practice guidelines [31]. CKD was defined as eGFR < 60 mL/min/1.73 m^2^ and/or an albumin-to-creatinine ratio > 30 mg/g measured on at least two occasions during 3 months or more, irrespective of the cause.

Family history of CVD, diabetes and hypertension was also recorded. Additionally, pre-existing non-fatal events of interest at baseline, including heart failure, coronary artery disease (CAD), cardiac arrhythmia, stroke and PAD, were defined by self-reported prior physician diagnosis of each comorbidity.

We also recorded possible hypertension-mediated organ damage (HMOD) and HRC at baseline and during the two years of follow-up. HMOD included stroke, left ventricular hypertrophy, abnormal ABI, and CKD. HRC included arteriosclerotic events (CAD, stroke and symptomatic PAD), heart failure, atrial fibrillation and CKD or its progression [1, 31]. CAD was defined as myocardial infarction (documented by elevated enzyme levels, with or without electrocardiography) or coronary artery revascularization using standard criteria [32]. Atrial fibrillation and left ventricular hypertrophy were ascertained by electrocardiography. Stroke was diagnosed as a persistent central neurological deficit lasting 24 h and unexplained by other causes. Diagnosis of heart failure was based on symptoms and/or signs (shortness of breath, peripheral oedema, pulmonary oedema/congestion by chest X‐ray), and dilated ventricle or poor left ventricular function evaluated by echocardiography. Finally, the presence of PAD at baseline was determined by a history of intermittent claudication, leg revascularization, amputation or an ABI ≤ 0.9. Intermittent claudication was defined as exertional leg pain relieved within 10 min by resting [7]. Additionally, AS was considered when ABI > 1.4.

### Cardiovascular health metrics

We used the American Heart Association definition of CVH based on 7 metrics: smoking status, physical activity, diet, body mass index, blood pressure, total cholesterol, and fasting blood glucose [19]. Each metric was categorized into three levels of poor, intermediate and ideal (Table S1). Consistent with the LS7 scoring approach, hypertensive patients on treatment who achieved their target levels for blood pressure (systolic blood pressure < 140 mmHg and diastolic blood pressure < 90 mmHg), cholesterol (total cholesterol < 200 mg/dl), and glucose (serum glucose < 126 mg/dl) were placed in the intermediate health category. Because we assessed a hypertensive population, no patients had an ideal blood pressure category. Thus, for each individual, a CVH score was created by recoding the 7 metrics as variables with 3 levels (poor, intermediate and ideal), with a score of 1, indicating the ideal status for each metric, and a score of 0 indicating intermediate and poor status; thus the CVH score could range from a minimum of 0 (indicating extremely poor CVH) to a maximum of 7 (reflecting ideal CVH).

### Measurement of ABI

The ABI was measured in all patients by trained technicians using an improved automated oscillometric device (MESI ABPI MD® system, Ljubljana, Slovenia). Agreement between oscillometric and conventional Doppler methods has been previously assessed [33]**.**

### Study outcomes

**Fig. 2:**
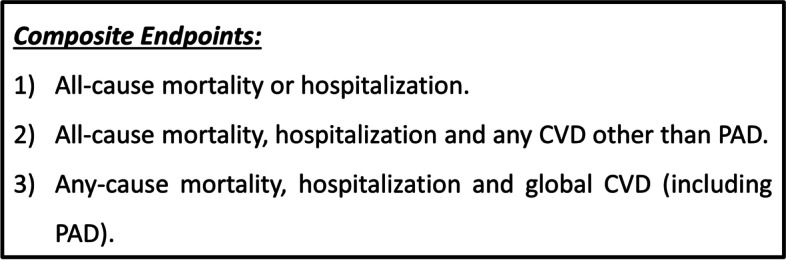
Composite endpoints assessed in the study. Abbreviations: CVD, cardiovascular disease; PAD, peripheral arterial disease

The primary outcome in our study was pre-existing or incident PAD (intermittent claudication, revascularization, amputation or ABI ≤ 0.9) as well as pre-existing or incident AS (ABI > 1.4). Additionally, the status of all patients was determined and data on mortality and hospitalizations were also obtained for the entire cohort at the end of follow-up. Thus, we generated three composite endpoints based on: 1) all-cause mortality or hospitalization; 2) all-cause mortality, hospitalization and any CVD other than PAD; and 3) any-cause mortality, hospitalization and global CVD (including PAD) (Fig. 2). We used any-cause hospitalization as an endpoint because the CVH metric has also been associated with multiple nonvascular conditions [34].

### Statistical analysis

All continuous variables with a normal distribution were expressed as the mean ± standard deviation. Continuous variables with a skewed distribution were presented as medians (interquartile ranges). Categorical variables were presented as percentages. Inter-group comparisons of quantitative variables were made by Student’s t-test or the Mann–Whitney U test as appropriate. Categorical variables were compared using the chi-square test or Fisher’s exact test as appropriate. The distribution of each LS7 component as poor, intermediate or ideal and the percentage of participants with zero to seven ideal goals were calculated. Additionally, the patients were stratified by ABI into three subgroups according to ABI measurement: 1) normal ABI (0.91 to 1.4); 2) ABI ≤ 0.9; and 3) ABI > 1.4 for comparisons. A cut-off value of ABI ≤ 0.9 has a high sensitivity (68–84%) and specificity (84–99%) for the diagnosis of PAD. In addition, a cut-off value of ABI > 1.4 indicates noncompressible arteries suggesting AS, as documented [10, 11]. We also calculated the prevalence of PAD (ABI ≤ 0.9) and subclinical arteriosclerosis (ABI > 1.4) by level of each LS7 factor. Multiple binary logistic regression models were used to assess the relationship between individual and cumulative CVH scores with the composite endpoints after adjusting for confounder variables. Models for each single component of the CVH metrics were adjusted for the other individual components of the healthy lifestyle score. Lastly, a multinomial logistic regression was used to assess the association between potential clinical risk factors and ABI measurements when considering the 3-level categorical variable (normal, ABI ≤ 0.9 and ABI > 1.4. All statistical analyses were performed using SPSS version 20.0 software (SPSS Inc., Chicago, IL), and a *P* < 0.05 was considered significant.

## Results

Of a total of 243 hypertensive patients, 57 (23.5%) had an abnormal ABI during the study, of which 16 (28%) had occlusive PAD (ABI ≤ 0.9) and 41 (72%) had AS (ABI > 1.4). Table 1 shows the baseline socio-demographic and clinical characteristics in patients with normal and abnormal ABI (ABI ≤ 0.9 and ABI > 1.4). Interestingly, the group with ABI ≤ 0.9 was found to be significantly associated with increased age, duration of hypertension, systolic blood pressure, number of antihypertensive and lipid-lowering drugs, high prevalence of diabetes and high prevalence of CKD and previous CVD other than PAD (CAD, stroke, left ventricular hypertrophy, heart failure and atrial fibrillation), and a higher mortality rate and hospitalizations than the rest of the patients. Likewise, the ABI ≤ 0.9 group also presented lower HDL-cholesterol levels and eGFR compared with other groups. Finally, a lesser number of ideal CVH metrics was also observed in the ABI ≤ 0.9 group. Other baseline clinical and socio-demographic and clinical characteristics were similar between the groups.

**Table 1:** Baseline characteristics of hypertensive patients with and without abnormal ABI

**Characteristics**^a^	**Normal ABI (*n* = 186)**	**ABI ≤ 0.9 (*n* = 16)**	**ABI > 1.4 (*n* = 41)**	***P* value**
**Age (y)**	68 ± 13	76.4 ± 10.9	68.2 ± 12.4	0.044
**Male (%)**	48.4	50	34.1	0.241
**Duration of hypertension (mo)**	160 ± 116	202.9 ± 112.4	119.9 ± 53.1	0.021
**Body mass index, (kg/m**^**2**^**)**	30.4 ± 5.1	31.7 ± 3.6	31 ± 4.5	0.732
**Waist-to-height ratio**	0.63 ± 0.08	0.67 ± 0.07	0.64 ± 0.07	0.180
**Systolic blood pressure, (mmHg)**	136.5 ± 17	144.6 ± 18.7	126 ± 14.7	< 0.001
**Diastolic blood pressure (mmHg)**	80.4 ± 9.7	75.9 ± 10.4	79.2 ± 8.9	0.166
**Fasting glucose (mg/dL)**	108.5 ± 30	126 ± 42.4	110.3 ± 55.3	0.188
**Total cholesterol (mg/dL)**	186.2 ± 38	173.4 ± 47.4	195.9 ± 31.4	0.108
**HDL cholesterol (mg/dL)**	51 ± 14	40.6 ± 11.4	52.5 ± 13.6	0.012
**LDL cholesterol (mg/dL)**	105 ± 35	93.2 ± 38.4	115.2 ± 31.6	0.085
**Log triglycerides (mg/dL)**	2.1 ± 0.2	2.2 ± 0.18	2.1 ± 0.2	0.152
**Diabetes (%)**	39.2	75	39	0.019
**Family history of CVD**^c^ **(%)**	58.1	68.8	65.9	0.500
**Family history of hypertension (%)**	78.5	75	87.8	0.357
**Family history of diabetes (%)**	53.8	81.3	56.1	0.105
**Education level (%)**				0.294
**Primary school or below**	70.4	93.8	68.3	
**Middle school**	22	6.3	26.8	
**High school or university**	7.5	0	4.9	
**Marital status (%)**				0.329
**Married or cohabiting**	61.3	62.5	61	
**Single or religious**	7	0	0	
**Divorced or widowed**	38.6	37.5	39	
**Alcohol consumption**^b^ **(%)**				0.134
**Low**	72.6	93.8	84.5	
**Moderate**	22	0	12.2	
**High**	5.4	6.3	2.4	
**Low income**^d^ **(%)**	81.7	85.4	100	0.433
**Nocturnal work (%)**	5.4	6.3	9.8	0.573
**eGFR (ml/min/1.73 m**^**2**^**)**	80 ± 20	61.2 ± 27.9	76.4 ± 20.6	0.002
**Log Albuminuria/Proteinuria (mg/g)**	1.3 ± 0.7	1.5 ± 0.6	1.0 ± 0.6	0.126
**HbA1C (%)**	5.9 ± 0.9	6.9 ± 1.4	6 ± 1.3	0.008
**C-reactive protein (mg/dL)**	3.5 ± 4.7	5 ± 6.6	4.2 ± 6.6	0.476
**CVD excluding PAD (%)**	42.5	81.3	39	0.008
**CKD (%)**	11.8	50	26.8	< 0.001
**Antihypertensive medication (%)**				
**• ACEI/ARA II**	93.5	86.7	90.2	0.512
**• Calcium antagonists**	29	56.3	24.4	0.051
**• Beta-blockers**	19.9	33.3	14.6	0.229
**• Vasodilators**	3.2	25	0	< 0.001
**• Diuretics**	48.4	66.7	56.1	0.301
**Number of antihypertensive (median IQR)**	2 (1–2)	3 (2–3)	2 (1–2)	< 0.001
**Lipid-lowering medication (%)**	55.4	81.3	43.9	0.039
**Left ventricular hypertrophy (%)**	20	56.3	17.1	0.002
**Hospitalization (%)**	24.7	62.5	19.5	0.002
**Death (%)**	2.2	25.0	2.4	< 0.001
**Ideal cardiovascular health metrics %**				0.020
**0–1**	39.2	50	38.3	
**2**	34	25.0	17.1	
**3**	15.6	18.8	26.8	
**4–5**	11.3	6.3	9.8	

*ABI* Ankle-branchial index includes values, *CVD* Cardiovascular disease, *CKD* Chronic kidney disease, *eGFR* Estimated Glomerular filtration rate, *PAD* Peripheral arterial disease, *ACEI/ARA II* Angiotensin-converting enzyme inhibitors or angiotensin-receptor antagonist, *IQR* Interquartile range. ^a^mean (SD) unless otherwise stated; ^b^low: < 10 g/d (men), < 5 g/d (women); moderate: 10–50 g/d (men), 5–10 g/d (women); high: ≥ 50 g/d (men), ≥ 10 g/d (women). ^c^*CVD* Cardiovascular disease, including myocardial infarction, heart failure, cardiac arrhythmia, stroke, and peripheral vascular disease; ^d^low income: < 25,000 €/y

### CVH metrics

**Fig. 3:**
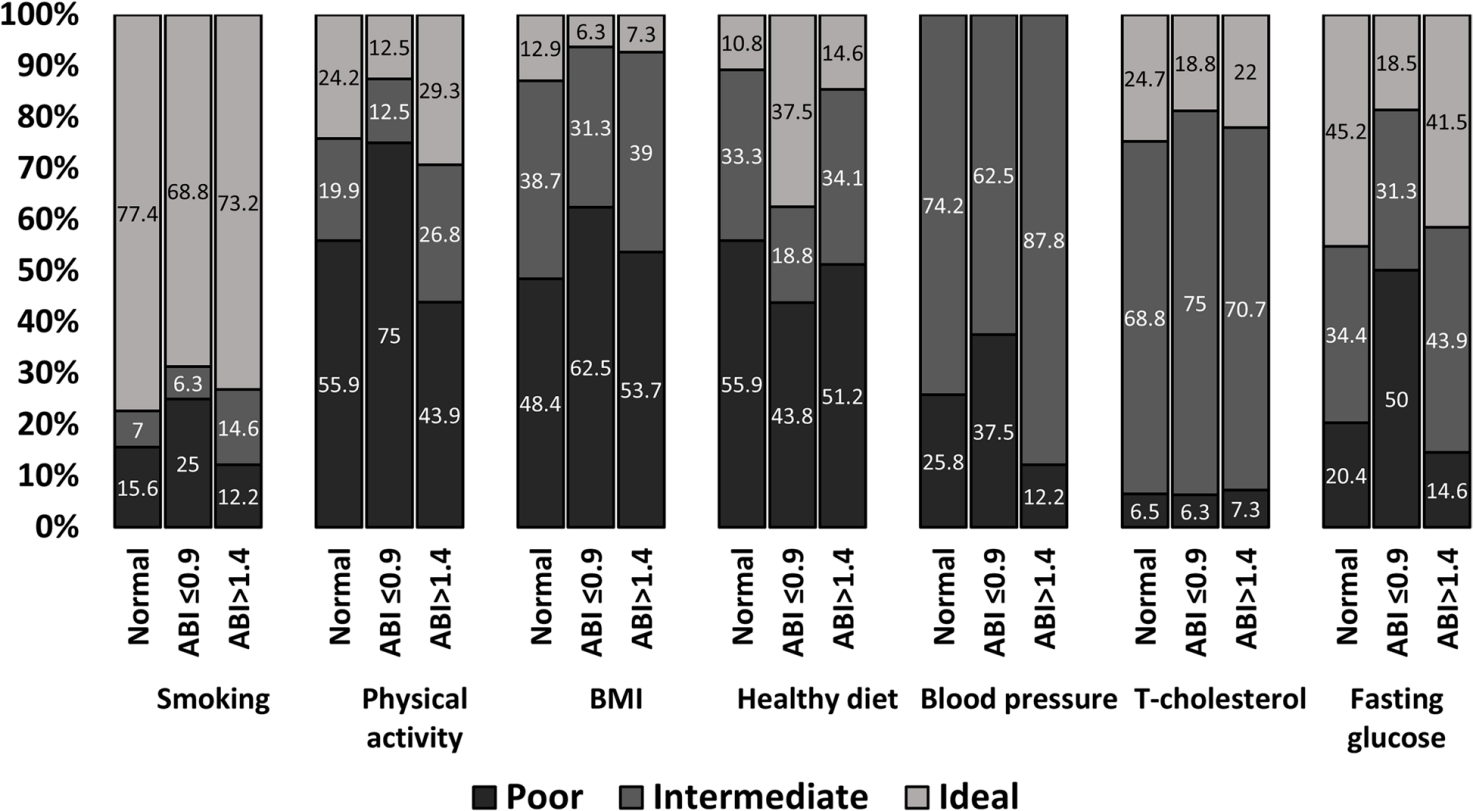
Prevalence of abnormal ABI associated with Life Simple 7 factor levels in the study populations. ABI (≤ 0.9 or > 1.4). Data are shown as percentage. Abbreviations: ABI, ankle-brachial index; BMI, body mass index

The median CVH score in the entire population was 2 (interquartile range 1–3) and the distribution of poor, intermediate and ideal LS7 components in patients with normal and abnormal ABI is presented in Fig. 3. Overall, smoking was the most common metric with ideal scores. A lesser proportion of ideal fasting glucose was observed in patients with ABI ≤ 0.9 (*P* = 0.034), whereas these patients showed a greater proportion of a healthy dietary pattern than the rest (*P* = 0.048). Due to the study design (all patients were hypertensive), no patient had an ideal blood pressure level because none presented untreated < 120/ < 80 mmHg. Only three patients presented 5 CVH metrics with ideal scores.

**Fig. 4:**
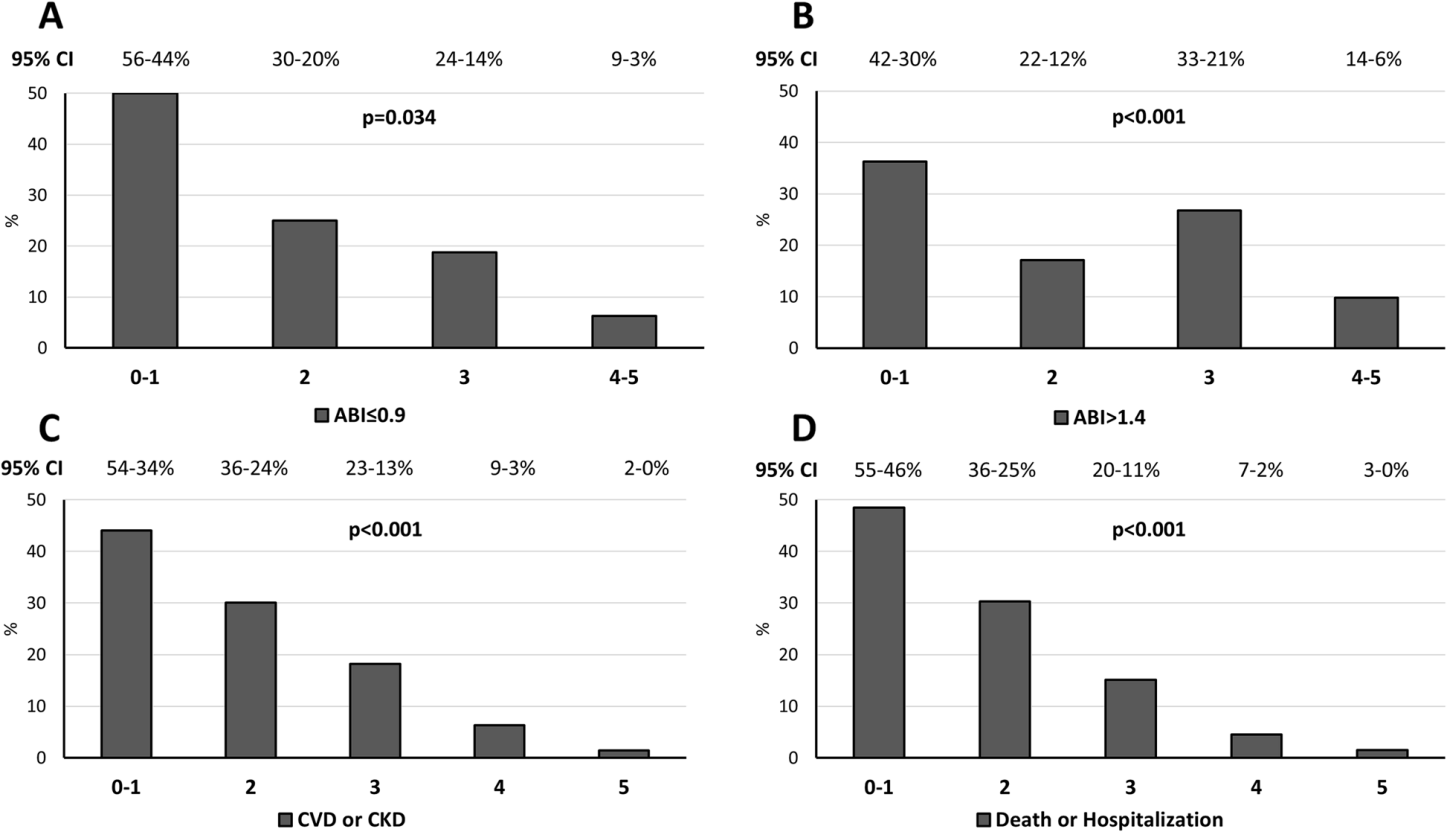
Baseline prevalence of ABI ≤ 0.9 (**A**) and ABI > 1.4 (**B**), pre-existing and incident CVD and/or CKD during follow-up (**C**) and mortality and hospitalization after two years of follow-up (**D**), according to the number of CVH metrics. Data are shown as percentage and 95% confidence interval. Chi-square test was used for comparisons. Abbreviations: ABI, ankle-brachial index; CKD, chronic kidney disease; CVH; cardiovascular health; CVD, cardiovascular disease

Figure 4A displays a significantly lower baseline prevalence of patients with ABI ≤ 0.9 as the number of ideal CVH metric scores increased. Similarly, the proportion of patients with pre-existing or incident CVD and/or CKD during follow-up (Fig. 4C) and the composite endpoint that included mortality and all-cause hospitalization (Fig. 4D) were also significantly lower as the number of ideal CVH scores increased. Lastly, Fig. 4B shows a significantly lower prevalence of ABI > 1.4 as the number of ideal CVH scores increased.

### Risk factors for abnormal ABI

**Fig. 5:**
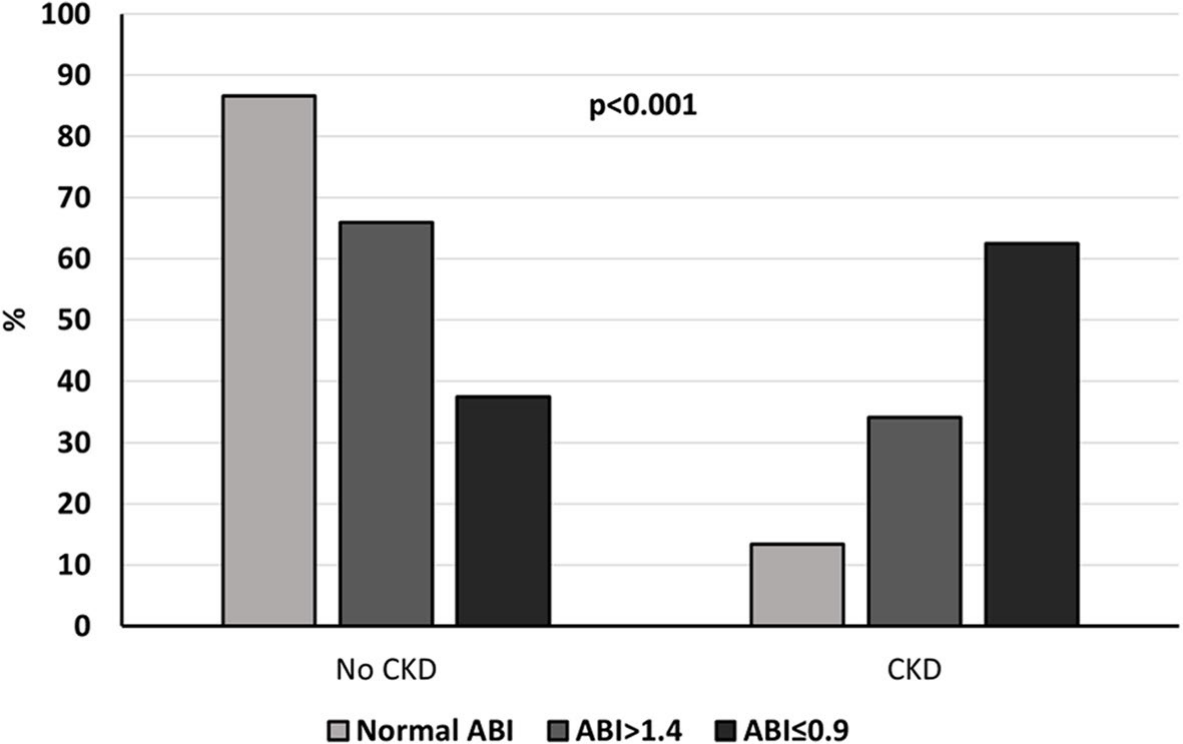
Proportion of CKD patients in subjects with abnormal ABI (≤ 0.9 or > 1.4) compared with the normal ABI group. Chi-square test was used for comparisons. Abbreviations: ABI, ankle-brachial index; CKD, chronic kidney disease

In an age-and sex-adjusted multivariate logistic regression analysis, neither the total CVH score nor other individual scores on the CVH metrics were associated with the presence of ABI ≤ 0.9 or ABI > 1.4. Of note, CKD (OR 6.7, 95% CI 2.04–21.8; *P* = 0.002) and diabetes (OR 4.5, 95% CI 1.3–15.2; *P* = 0.014) were significantly associated with an ABI ≤ 0.9. Likewise, when the presence of ABI > 1.4 was considered as the dependent variable (excluding patients with ABI ≤ 0.9), CKD was also an independent risk factor for AS (OR 3.3, 95% CI 1.4–7.8; *P* = 0.007), but not diabetes. As a consequence, a higher proportion of CKD patients was detected among patients with abnormal ABI (≤ 0.9 or > 1.4) compared with the normal ABI group (Fig. 5), and a significant correlation was observed between GFR and ABI measurement (*r* = 0.213; *P* = 0.01). Finally, in a multinomial logistic regression analysis, GFR evaluated by MDRD, systolic blood pressure and ideal physical activity and diet were significantly related to ABI measurements (Table 2).

**Table 2:** Factors associated with ABI measurements^a^

Variable	ABI ≤ 0.9			ABI > 1.4		
	**OR**	**95% CI**	***P***	**OR**	**95% CI**	***P***
**Male sex**	2.550	0.695–9.357	0.158	0.556	0.256–1.209	0.139
**Age, y**	1.032	0.976–1.091	0.270	0.997	0.966–1.029	0.837
**eGFR, mL/min**	0.967	0.941–0.994	0.018	0.989	0.969–1.009	0.279
**Systolic blood pressure, mmHg**	1.036	1.001–1.071	0.041	0.959	0.936–0.982	0.001
**Ideal health diet**	3.107	1.312–7.358	0.010	1.059	0.642–1.746	0.823
**Ideal physical activity**	0.363	0.138–0.954	0.040	1.565	1.000–2.449	0.050

*ABI* Ankle-brachial index, *eGFR* Estimated glomerular filtration rate

^a^Multinomial logistic regression model to predict ABI measurements. Dependent variable (outcome) consisted of three categories: 0 = Normal (reference), 1 = ABI ≤ 0.9 and 2 = ABI > 1.4

### Outcome follow-up

Table 3 displays HMOD and HRC at baseline and during the two-year follow-up, including atherosclerotic events (CAD, stroke and PAD), heart failure, atrial fibrillation, left ventricular hypertrophy and CKD. We recorded a total of 264 cases of cardiovascular conditions (HMOD and HRC) in 243 patients, of which 225 were collected in the initial assessment and 39 occurred during the follow-up. No patients had PAD during the two-year follow-up. We also recorded 9 deaths: 6 attributed to CVD, 2 due to cancer and 1 due to infectious complication. Of the 9 patients who died, 4 belonged to the ABI ≤ 0.9 group, one to the ABI > 1.4 group and the others had a normal ABI (*P* < 0.001). Additionally, a total of 64 patients had any-cause hospitalization during follow-up. Table 4 shows the ORs and 95% CIs of the composite endpoints based on each health and behaviour factor adjusted for covariates, including CVH metrics. Using mortality and hospitalization as the dependent variable (Model 1), the presence of CKD and occlusive PAD (ABI ≤ 0.9) were significantly associated with the composite endpoint. When the composite endpoint used as the dependent variable was death, hospitalization or CVD other than PAD (Model 2), it was found that age, sex, diabetes and CKD were significantly associated with unfavourable outcomes but not other individual metrics or the total CVH score. Finally, when any-cause death, hospitalization or global CVD (including PAD) was entered as the dependent variable (Model 3), age, sex, CKD and ideal cholesterol were associated with outcome after adjusting for confounders.

**Table 3:** Hypertension-mediated organ damage and hypertension-related comorbidities at baseline and during follow-up^a^

	**Baseline**	**Follow-up**	**Global**
**Ischemic heart disease, (n)**	14.2 (32)	10.3 (4)	13.6 (36)
**Stroke (n)**	6.7 (15)	18 (7)	8.3 (22)
**Peripheral arterial disease**^b^ **(n)**	7.1 (16)	-	6.1 (16)
**Atrial Fibrillation (n)**	22.2 (50)	28.2 (11)	23.1 (61)
**Left ventricular hypertrophy (n)**	23.6 (53)	12.8 (5)	22 (58)
**Heart failure (n)**	8 (18)	10.3 (4)	8.3 (22)
**Chronic kidney disease (n)**	18.2 (41)	20.5 (8)	18.6 (49)
**Total**	225	39	264

Data are provided as percentage

^a^Hypertension-mediated organ damage includes stroke, left ventricular hypertrophy, abnormal ankle-brachial index and CKD; Hypertension-related comorbidities include stroke, coronary artery disease, heart failure, atrial fibrillation, peripheral vascular disease and CKD or its progression

^b^Peripheral arterial disease was defined as intermittent claudication, amputation, revascularization of legs or ankle-brachial index ≤ 0.9

**Table 4:** Risk factors associated with death and hospitalization (Model 1), death, hospitalization or CVD other than PAD (Model 2) and death, hospitalization or CVD (Model 3)

	**OR**	**95% CI**	***P* value**
**Model 1**^a^			
Age	1.02	0.99–1.05	0.121
Male sex	1.84	0.99–3.42	0.053
Diabetes	1.67	0.90–3.09	0.103
CKD	2.31	1.03–5.18	0.043
ABI ≤ 0.9	4.13	1.26–13.47	0.019
ABI > 1.4	0.66	0.27–1.61	0.364
**Model 2**^b^			
Age	1.04	1.02–1.07	0.001
Male sex	2.71	1.51–4.89	0.001
Diabetes	2.43	1.35–4.37	0.003
CKD	4.61	1.69–12.56	0.003
ABI ≤ 0.9	1.97	0.47–8.20	0.353
ABI > 1.4	0.77	0.35–1.69	0.513
**Model 3**^c^			
Age	1.03	1.01–1.06	0.015
Male sex	2.09	1.15–3.78	0.015
Diabetes	1.79	0.98–3.28	0.058
CKD	6.68	1.87–23.88	0.003
Ideal cholesterol	0.44	0.23–0.87	0.018

Dependent variable:

^a^Death and/or hospitalization

^b^Death and/or hospitalization and/or CVD other than PAD

^c^Death and/or hospitalization and/or CVD

*ABI* Ankle-brachial pressure index, *CKD* Chronic kidney disease, *CVD* Cardiovascular disease, *PAD* Peripheral arterial disease

## Discussion

### Summary of findings

Our observational study showed that the prevalence of ideal CVH LS7 goals in hypertensive patients from an urban population was low. A lower prevalence of ABI ≤ 0.9, and the composite endpoints CVD or CKD as well as death and hospitalization was found as the number of ideal CVH scores increased. Finally, age, male sex, diabetes, CKD, ABI ≤ 0.9 and ideal cholesterol, but not other individual CVH metrics, were significantly associated with unfavourable outcomes.

### Comparison with previous studies and risk factors

The concept of ideal CVH appeared in 2010 in the core of the American Heart Association to improve the health of the USA population [19]. Since then numerous observational studies have demonstrated that a higher number of ideal LS7 components is associated with more favourable outcomes [20, 25, 27, 28, 35–38]. In addition, ideal goals in CVH metrics have also been related with lower rates of PAD and subclinical atherosclerosis in multiple large cross-sectional and longitudinal observational studies in different populations [7, 21, 26, 27, 39, 40], but in America or China only 0.1% of the population were found to have all 7 ideal goals in CVH metrics [41–45]. In particular, Wang et al. found that CVH metrics is strongly associated with the new occurrence of PAD in a large observational study using a standard method to detect the presence of PAD [26]. In African Americans, Collins et al. also observed that a lower number of ideal LS7 metrics is associated with the prevalence of PAD [46], and the same conclusions were reported by Garg et al. [7]. Similar conclusions were also reached in the general Chinese, Spanish, Finnish and Australian populations [23, 27, 47, 48]. As an example, ideal cardiovascular health has been inversely associated with subclinical atherosclerosis, including carotid atherosclerosis and PAD, in large prospective studies [27, 48, 49]. In addition, the presence of more ideal CVH metrics was not only associated with AS, but also independently predicted annual change in AS in the study performed by Sang et al. [50]. In all these studies, the prevalence of ideal CVH metrics was extremely low (< 3%), as seen in the current study.

These findings are in accordance with our study and highlight the importance of assessing the effect of ideal CVH on both clinical and subclinical CVD in a high-risk population like that of our study. Accordingly, besides a low ideal CVH prevalence, we observed in our study a lower prevalence of ABI ≤ 0.9, and a lower proportion of patients with CVD and/or CKD, and death and hospitalization decreased as the number of ideal goals on the CVH metrics increased. Likewise, we also observed a lower proportion of patients with ABI > 1.4 as CVH metrics increased gradually.

A poor cardiovascular health status, evaluated through the LS7 metrics, could contribute to the life-threatening complications, including PAD, in hypertensive patients. The positive assessment of these seven estimates has been associated with a decrease in the risk of CVD and a lower rate of global and cardiovascular mortality in multiple large observational studies [7, 20, 21, 23, 28, 44]. Indeed, individuals with 5–7 ideal CVH metrics have a much lower rate of CVD and cardiovascular mortality, as well as all-cause mortality, than those with 0–1 ideal CVH metrics. Conversely, those with a lower number of ideal LS7 metrics have an increased risk of hypertension-mediated comorbidities, such as PAD, CAD, stroke and left ventricular hypertrophy, and any-cause mortality [20, 28, 45]. Additionally, both the total LS7 score and individual CVH metrics have been associated with these unfavourable outcomes.

In consonance with previous reports, occlusive PAD was associated in the current study with unfavourable outcomes, and only ideal total cholesterol showed a significant association with the composite endpoint that included CVD, mortality or hospitalization after adjusting for confounders such as lipid-lowering medication, which reinforces the possible association between a poor lipid profile and unfavourable outcomes. However, the total CVH score and other individual metrics other than ideal cholesterol did not significantly associate with the unfavourable endpoints in the multivariate analysis. The reasons for these findings are not clear, but we speculate that some explanations might account for our findings. Firstly, only a few patients reached the score of 5 ideal health factors and no patient reached 6 or 7 ideal goals, though an ideal level of blood pressure was impossible due to the study design. Secondly, we studied mostly an elderly population with a high prevalence of other well-known vascular risk factors such as body mass index > 30 kg/m^2^ (50.2%), diabetes (41.6%) or the presence of CKD (16%), which could have contributed to the scarcity of healthy factors and healthy behaviours in our patients. It is plausible to think that, given the study design of consecutive patient selection, these high-risk patients were less likely to possess ideal CVH metrics, leading to an increased risk of PAD and poorer outcomes. In other words, the excess risk of PAD among older hypertensive patients may be explained by the clustering of other cardiovascular risk factors associated with hypertension, such as diabetes or the treatment itself. This suggests that recommendations for the general population, included in the CVH metric, might not apply to an elderly hypertensive population with a high burden of traditional and non-traditional vascular risk factors, as in our study. As an example, in recent observational studies the LS7 diet and body mass index components, as well as the optimal blood pressure, showed a lack of association with unfavourable outcomes in patients with advanced CKD [29, 51, 52], perhaps because recommendations for renal patients, mainly when other relevant cardiovascular risk factors coexist, conflict to a certain extent with those for the general population. In other words, the explanation for the conflicting results may be that they were not affected so much by residual confounding from chronic conditions often found in older cohorts with a high burden of comorbidities like in our population.

PAD indicates atherosclerosis in other artery territories. Thus, it is not surprising that ABI was associated with unfavourable outcomes in the current study. Additionally, diabetes and CKD are two strong cardiovascular risk factors for comorbidities and death. In particular, renal patients, especially those with diabetes, have an extraordinarily high cardiovascular mortality risk [25, 53–55], which increases as the GFR worsens. Indeed, in our study these two risk factors were also associated with the more severe kind of PAD (ABI ≤ 0.9) and mortality, but not the CVH score. CKD was significantly more frequent in patients with an abnormal ABI (ABI ≤ 0.9 or ABI > 1.4) compared with the rest. Moreover, the GFR showed a significant correlation with ABI measurements in our multivariate analysis and it was an independent risk factor for ABI measurements. Additionally, renal patients tended to have a higher blood pressure (data not shown). Similarly, diabetes was also more frequent in the ABI ≤ 0.9 group compared with the normal ABI or ABI > 1.4 groups. These cut-off values for ABI interpretation have been previously proposed for the diagnosis of both PAD and AS, and represent a reasonable standardized categorization in these patients [11]. Taken together, it is plausible to think that CKD (or decreased GFR) could be intermediates between health behaviours and health factors and an abnormal ABI, especially in elderly hypertensive patients. The fact that in a large observational study the association between a better cardiovascular profile and a better healthy profile was lessened and not present any more after adjustment for GFR and albuminuria supports this hypothesis. Further large observational studies are needed to elucidate this concern in hypertensive patients with an increased cardiovascular burden.

AS is considered an additive damage to the arterial wall among traditional cardiovascular risk factors, including hypertension, that occurs with ageing. Although previous observational studies have found an inverse relationship between CVH status and AS [23, 50], we did not. We hypothesized, thus, that the protective effect of ideal CVH metric on AS could have been underestimated by the confluence of multiple risk factors in an elderly hypertensive population like in our study. Arterial aging-related structural and functional changes may be accelerated by hypertension and diabetes. In addition, the proposed CVH metrics include seven ideal components, and no patient had ideal blood pressure control given the design of the study. Taken together, this could well account for the absence of a relationship between CVH metrics and AS in the current study.

### Implications

Given that hypertension-mediated target end-organ damage increases the risk of overt CVD, including PAD, our findings could have considerable importance as they highlight the need for early adequate blood pressure control in order to prevent hypertension-related life-threatening complications such as CKD or PAD. Additionally, identifying other modifiable risk factors for CKD such as obesity or diabetic status could be more relevant, especially in elderly hypertensive patients with a high burden of cardiovascular risk factors.

### Limitations and strengths

Our main limitation is that we assessed a relatively small sample size of hypertensive patients and the ability to determine the association between some variables might therefore be limited. Likewise, in any observational study, residual confounding cannot be excluded. Results of our study may not be generalizable to other populations with a different context, age, race and lifestyle culture. Our patients were mostly old, had multiple comorbidities and came from an urban population, which may have also led to selection bias. Additionally, due to the self-reported nature of the data collected, social desirability bias was also likely. Furthermore, we did not collect data on adherence to anti-hypertensive drugs. This could also underestimate the impact of CVH metrics on outcome. Left ventricular hypertrophy was mostly ascertained by electrocardiography. In addition, we did not assess routinely the presence of hypertensive retinopathy. Thus, the prevalence of HMOD could have been underestimated. Perhaps future studies with a larger sample size and a more prolonged follow-up are needed in this particular population. Finally, with the data from this study we were unable to evaluate whether changes in CVH metrics really reduce PAD risk, as previously reported in other cardiovascular entities [56]. In fact, an improvement from a poor to intermediate or ideal LS7 score was also associated with a lower risk of CVD such as CAD, stroke and heart failure, but PAD was not assessed in that cohort study. Although preventive and therapeutic strategies were implemented during follow-up in our patients with lower ideal CVH metrics to optimise their ideal LS7 score (increasing anti-hypertensive medication, providing ideal diet and advising physical activity, and using modern medication to treat diabetes and dyslipidaemia, among others), we did not assess whether changes in CVH metrics really decrease hypertension-related complications and other unfavourable outcomes.

Despite these limitations, our study also has some strengths. We performed a longitudinal observational study and collection of baseline data was broad and complete using a standardized questionnaire. There were no missing data in the current study. In addition, we followed strictly not only the recommendations of the 7 American Heart Association ideal metrics as previously reported [19], but also recorded other social-demographic data such as family income, nocturnal work, education level or alcohol consumption, which could have a negative influence on CVH metrics.

## Conclusions

This study shows that the prevalence of ideal CVH was low in hypertensive patients from an urban population with a high burden of known cardiovascular risk factors. An inverse relationship between CVH score and ABI ≤ 0.9 and outcomes (CVD, CKD, death or hospitalization) was observed. Older age, male gender, diabetes, CKD and ABI ≤ 0.9 were positively associated with worse outcomes, while ideal cholesterol levels were inversely associated. Thus, stronger efforts at health promotion and intervention studies aimed to booster adoption of ideal CVH may improve outcomes among community-based hypertensive patients. In this line, early detection of hypertension (with walk-in clinics, for example) and adequate blood pressure treatment, control of obesity providing ideal diet and advising physical activity, stopping smoking and using modern therapeutic strategies to manage diabetes and dyslipidaemia can attenuate hypertension-related life-threatening complications, including PAD, mainly in elderly patients.

## Supplementary Information

**Additional file 1: Table S1.** Definitions for the three category indicators of CVH (poor, intermediate and ideal), as per American Heart Association specifications.

## Data Availability

Data are available on request due to privacy restrictions. The data presented in this study are available on request from the corresponding author. In compliance with Spanish Organic Law 15/1999, the data are not publicly available.

## Abbreviations

ABI: Ankle-brachial index
AS: Arterial stiffness
CAD: Coronary artery disease
CKD: Chronic kidney disease
CVD: Cardiovascular disease
CVH: Cardiovascular health
HMOD: Hypertension-mediated organ damage
HRC: Hypertension-related comorbidities
LS7: Life´s Simple 7
PAD: Peripheral artery occlusive disease
