# Supplementary material for: Abnormal ankle-brachial index, cardiovascular risk factors and healthy lifestyle factors in hypertensive patients: prospective cohort study from a primary care urban population
Source: BMC Prim Care. 2022 Sep 9;23:232. doi: 10.1186/s12875-022-01837-1 (PMC9463763; doi:10.1186/s12875-022-01837-1)
Supplement: Supplementary file 1 — Additional file 1: Table S1. Definitions for the three category indicators of CVH (poor, intermediate and ideal), as per American Heart Association specifications. [file 12875_2022_1837_MOESM1_ESM.docx]

**Table S1**. Definitions for the three category indicators of CVH (poor, intermediate and ideal), as per American Heart Association specifications

| **Cardiovascular health-LS7 indicators** | **Poor** | **Intermediate** | **Ideal** |
| --- | --- | --- | --- |
| Smoking | Current smoker | Former smoker who quit ≤12 mo. ago | Never |
| Total cholesterol (mg/dL) | ≥240 | 200-239 or treated to control | <200 without medication |
| Blood pressure (mmHg) | SBP ≥140 or DBP ≥90 | SBP 120 to 139 or DBP 80 to 89 or treated to control | <120/<80 without medication |
| Fasting plasma glucose (mg/dL) | ≥126 | 100 to 125 or treated to control | <100 without medication |
| Body mass index (kg/m^2^) | ≥30 | 25 to 29.9 | <25 |
| Physical activity | None | 1-149 min/week moderate intensity or  1-74 vigorous intensity | ≥150 min/week moderate intensity or ≥75 min/week vigorous intensity |
| Healthy diet score* | 0-1 components | 2-3 components | 4-5 components |

CVH, cardiovascular health; SBP, systolic blood pressure; DBP, diastolic blood pressure; LS7, Life’s Simple 7.

*Fruits and vegetables ≥4-5 cups/day; fish ≥2-3 servings/week; fibre-rich whole grains ≥3 servings/day; sodium <1500 mg/day; sugar-sweetened beverages ≤450 kcal/week.
